# A MIF‐Derived Cyclopeptide that Inhibits MIF Binding and Atherogenic Signaling via the Chemokine Receptor CXCR2

**DOI:** 10.1002/cbic.202000574

**Published:** 2020-11-30

**Authors:** Christine Krammer, Christos Kontos, Manfred Dewor, Kathleen Hille, Beatrice Dalla Volta, Omar El Bounkari, Karin Taş, Dzmitry Sinitski, Markus Brandhofer, Remco T. A. Megens, Christian Weber, Joshua R. Schultz, Jürgen Bernhagen, Aphrodite Kapurniotu

**Affiliations:** ^1^ Division of Vascular Biology Institute for Stroke and Dementia Research (ISD) LMU Klinikum Ludwig-Maximilians-Universität (LMU) Feodor-Lynen-Straße 17 81377 Munich Germany; ^2^ Division of Peptide Biochemistry TUM School of Life Sciences Technische Universität München (TUM) Emil-Erlenmeyer-Forum 5 85354 Freising Germany; ^3^ Institute of Biochemistry and Molecular Cell Biology University Hospital RWTH Aachen University Pauwelsstrasse 30 52074 Aachen Germany; ^4^ Institute for Cardiovascular Prevention LMU Klinikum Ludwig-Maximilians-Universität (LMU) Pettenkoferstrasse 8a and 9 80336 Munich Germany; ^5^ Munich Cluster for Systems Neurology (SyNergy) Feodor-Lynen-Straße 17 81377 Munich Germany; ^6^ Munich Heart Alliance Biedersteiner Straße 29 80802 Munich Germany; ^7^ Cardiovascular Research Institute Maastricht (CARIM) Maastricht University Universiteitssingel 50 6229 Maastricht (The Netherlands; ^8^ Carolus Therapeutics, Inc. 5626 Oberlin Drive 92121 San Diego CA USA; ^9^ Present address: Moderna Therapeutics, Inc. 200 Technology Square Cambridge MA 02139 USA

**Keywords:** alanine scanning, atherosclerosis, chemokine receptors, cyclic peptides, macrophage migration inhibitory factor

## Abstract

Macrophage migration inhibitory factor (MIF) is an inflammatory cytokine and atypical chemokine with a key role in inflammatory diseases including atherosclerosis. Key atherogenic functions of MIF are mediated by noncognate interaction with the chemokine receptor CXCR2. The MIF N‐like loop comprising the sequence 47–56 is an important structural determinant of the MIF/CXCR2 interface and MIF(47–56) blocks atherogenic MIF activities. However, the mechanism and critical structure–activity information within this sequence have remained elusive. Here, we show that MIF(47–56) directly binds to CXCR2 to compete with MIF receptor activation. By using alanine scanning, essential and dispensable residues were identified. Moreover, MIF(cyclo10), a designed cyclized variant of MIF(47–56), inhibited key inflammatory and atherogenic MIF activities *in vitro* and *in vivo/ex vivo*, and exhibited strongly improved resistance to proteolytic degradation in human plasma *in vitro*, thus suggesting that it could serve as a promising basis for MIF‐derived anti‐atherosclerotic peptides.

## Graphical abstract

**The specific targeting** of the interaction between the atypical chemokine MIF and its receptor CXCR2, and its atherosclerosis‐promoting activity in a tailored peptide‐based approach is illustrated. Based on a structure–activity analysis of short N‐like loop‐derived MIF peptides, the cyclic MIF peptide analogue MIF(cyclo10) was identified as a promising candidate that blocks MIF/CXCR2 and counteracts MIF's inflammatory and pro‐atherogenic activity.

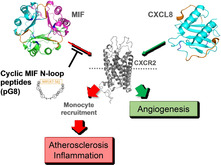

## Introduction

Atherosclerosis is a lipid‐triggered chronic inflammatory disease of the large‐ and medium‐sized arteries that is characterized by the infiltration of leukocytes, vascular inflammation, the formation of lipid‐rich intimal plaques, and massive tissue remodeling and necrotic core build‐up. These plaques can eventually rupture leading to thrombus formation, resulting in detrimental cardio–vascular diseases such as myocardial infarction and ischemic stroke.^[1]^ Chemokines and their receptors play an important role in atherogenesis by orchestrating the leukocyte infiltration cascade and promoting vascular inflammation.^[2]^ Forty‐nine human chemokines and 19 G protein‐coupled receptor (GPCR)‐type chemokine receptors have been described and chemokines can be classified into the four subclasses C−, CC−, CXC− and CX_3_C−, based on the position and number of conserved cysteine residues at their N‐terminus. CXC chemokines are further sub‐classified into ELR+ and ELR‐ chemokines. ELR+ chemokines carry the tripeptide motif glutamic acid‐leucine‐arginine (ELR) N‐terminal to the first cysteine residue.^[3]^ Interactions within the chemokine ligand/receptor network are characterized by redundancy and promiscuity, with many chemokines binding to more than one receptor and *vice versa*.^[4]^ Yet, responses are typically highly specific, a principle that has been described by the terms “ligand, receptor, or tissue bias”.^[5]^ According to the classical model, chemokine binding to its receptor follows a two‐site mechanism. In this paradigm, the chemokine N‐loop interacts with the N‐terminal domain of the receptor (site 1), while the chemokine N‐terminus binds to a nonconsecutive interface consisting of extracellular loops (ECLs) and/or portions of the transmembrane region of the receptor (site 2). Site 1 interaction mainly determines the binding affinity and receptor selectivity, while site 2 interaction is important for receptor activation.[^4^, ^5^]

The complexity in the chemokine ligand/receptor network is further complicated by atypical chemokines (ACKs) such as macrophage migration‐inhibitory factor (MIF) or human β‐defensins (HBDs). ACKs do not belong to any of the four chemokine classes, but mimic certain structural properties of chemokines and engage in high‐affinity binding to classical chemokine receptors.^[6]^ MIF is a prototypical ACK and an evolutionarily conserved pleiotropic inflammatory cytokine. Owing to this capacity, it is a pivotal mediator of acute and chronic inflammatory diseases, including atherosclerosis.[^6^, ^7^] A pro‐atherogenic role of MIF is evident from numerous preclinical studies involving neutralizing MIF antibodies and *Mif* gene depletion in the context of atherogenic *Apoe* ^*−/−*^ or *Ldlr* ^*−/−*^ mice, as well as clinical correlations in atherosclerotic patients.[^6^, ^7^] MIF broadly increases vascular inflammation and specifically promotes the atherogenic recruitment of monocytes and T cells through noncognate interaction with the chemokine receptors CXCR2 and CXCR4, respectively.^[7a]^ Thus, MIF is an interesting target for drug development in atherosclerosis and other chronic inflammatory conditions.^[6]^ In fact, antibody‐ and small‐molecule‐based approaches have been developed against MIF. However, these are predicted to lack specificity in atherosclerotic diseases, because they have been shown to fully neutralize MIF and thus not only target atherogenic interactions between MIF and its chemokine receptors, but could interfere with the homeostatic capacity of MIF and compromise binding to the cardioprotective receptor CD74.[^6^, ^8^]

As the MIF/CXCR2 axis is a major pro‐atherogenic axis and to better fine‐tune the targeting of MIF/receptor interactions, we focused on peptide‐based strategies to specifically block the interaction between MIF and CXCR2. Such a strategy also could avoid affecting interactions between CXCR2 and its *bona fide* ligand CXCL8.^[9]^ A prerequisite for the development of such inhibitors is a detailed knowledge of the structural determinants of the receptor/ligand interface. We showed that the interaction between MIF and CXCR2 follows a two‐site binding mechanism and identified critical binding motifs. Site 1 consists of the MIF N‐like loop, comprising residues 47–56 and the receptor N‐domain together with ECL1 and parts of ECL2, while site 2 is comprised of the pseudo‐(E)LR motif of MIF and parts of ECL2 and ECL3.[^7b^, ^10^]

## Results and Discussion

We have tested the capacity of MIF(47–56)‐derived peptides to function as MIF/CXCR2‐specific inhibitors, have performed a structure‐activity analysis of this sequence region, and have developed a cyclized variant with anti‐atherogenic capacity.

**Figure 1:**
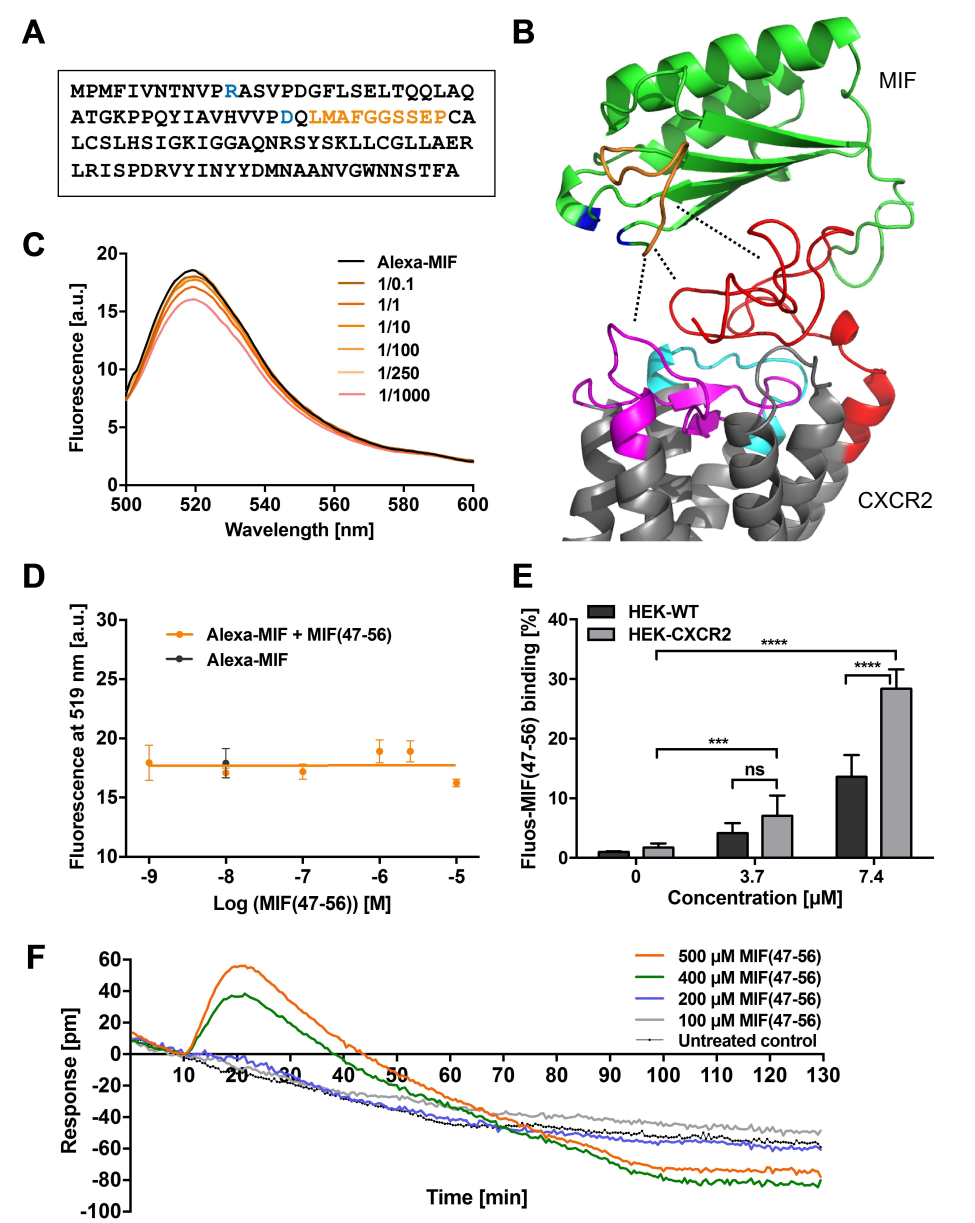
Interaction of peptide MIF(47–56) with CXCR2. A) Sequence of human MIF. Residues of the pseudo‐(E)LR motif (blue) and MIF sequence 47–56 (orange) are highlighted. B) Structure model of the complex between human MIF (green; PDB ID: 3DJH:A) and CXCR2 (gray; structure predicted by Phyre^2^ ^[11]^ and as predicted by protein–protein docking in PatchDock/FireDock for visualization purposes^[12]^). The MIF sequence 47–56 is highlighted in orange, the pseudo‐(E)LR motif is depicted in blue; for CXCR2, the N‐domain (red), and parts of ECL1 (cyan) and 2 (magenta), that is, the regions that have been suggested to contribute to the interface with MIF, are also color‐coded. Dotted lines indicate interaction contact points. C), D) Fluorescence spectroscopic titrations of Alexa‐488‐labeled rMIF (Alexa‐MIF, 10 nM) with increasing concentrations of MIF(47–56) (0.1‐ to 1000‐fold molar excess). C) Fluorescence spectra of the various mixtures and of Alexa‐MIF alone recorded between 500 and 600 nm are shown. D) The fluorescence emission at 519 nm was plotted against the peptide concentration (three titration experiments, mean±SD). E) Binding of Fluos‐MIF(47–56) (3.7 or 7.4 μM) to CXCR2, stably expressed on HEK293 cells in comparison to non‐transfected wild‐type HEK293 cells. The mean fluorescence intensity (MFI) was measured by flow cytometry and intensities normalized to the signal of non‐transfected control cells (*n*=4–7, mean±SD). Statistical significance is indicated: *** *P*<0.001, **** *P*<0.0001; ns, not significant; WT, wild type. F) Detection of MIF(47–56) binding to CXCR2 by label‐free dynamic mass redistribution (DMR) technology. HEK293‐CXCR2 transfectants were treated with MIF(47–56) (at 100, 200, 400, 500 μM, as indicated), and cellular responsiveness as a measure of binding was recorded at 30 s intervals for a total of 120 min.

MIF sequence 47–56 (Figure 1A) contributes to the site 1 binding area between MIF and CXCR2 (Figure 1B) and MIF‐derived peptide MIF(47–56) inhibits MIF‐stimulated inflammatory activities,^[10]^ but the exact mechanism underlying this inhibitory effect has remained unclear. We wanted to determine whether MIF(47–56) directly binds to MIF (in its monomeric or trimeric form) or whether it competes with MIF/CXCR2 binding at the interface by direct interaction with CXCR2. Fluorescence spectroscopic titrations of Alexa‐488‐labeled recombinant MIF (Alexa‐MIF) with increasing concentrations of MIF(47–56) revealed that the peptide does not directly bind to MIF (Figure 1C, D). In line with this notion, fluorescently labeled MIF(47–56) (Fluos‐MIF(47–56)) specifically bound to full‐length CXCR2, when stably expressed on the surface of HEK293 cells and compared to non‐transfected control cells (Figure 1E). Moreover, MIF(47–56) was found to bind to HEK293‐CXCR2 transfectants as determined by changes of the resonant wavelength Δpm using label‐free dynamic mass redistribution (DMR) technology (Figure 1F). These data suggest that MIF(47–56) does not bind to MIF but directly interacts with cell surface‐expressed CXCR2.

To pinpoint the residues that predominantly determine the inhibitory effect of MIF(47–56) and in particular to map sequence positions that could be dispensable and suitable for substitutions towards the design of second generation peptide variants, we performed position‐selective alanine‐scanning, replacing residues 47, 48, and 53–56 by Ala (Table 1). Alanine mutants for residues 49 and 50 were not generated, as residue 49 is an Ala in the native sequence and Phe‐50 is involved in subunit interactions within the MIF trimer.^[13]^ Also, we did not generate mutants for Gly‐51 and Gly‐52, as such mutations would likely interfere with loop flexibility and function.^[10]^

**Table 1:** Sequences of MIF(47–56) peptide variants as generated by alanine‐scanning.

Peptide variant	Sequence
MIF(47–56)	LMAFGGSSEP
MIF(47–56/L47A)	AMAFGGSSEP^[a]^
MIF(47–56/M48A)	LAAFGGSSEP
MIF(47–56/S53A)	LMAFGGASEP
MIF(47–56/S54A)	LMAFGGSAEP
MIF(47–56/E55A)	LMAFGGSSAP
MIF(47–56/P56A)	LMAFGGSSEA

[a] Substitution is indicated in peptide name, and alanine substitutions are highlighted in red in the sequence.

**Figure 2:**
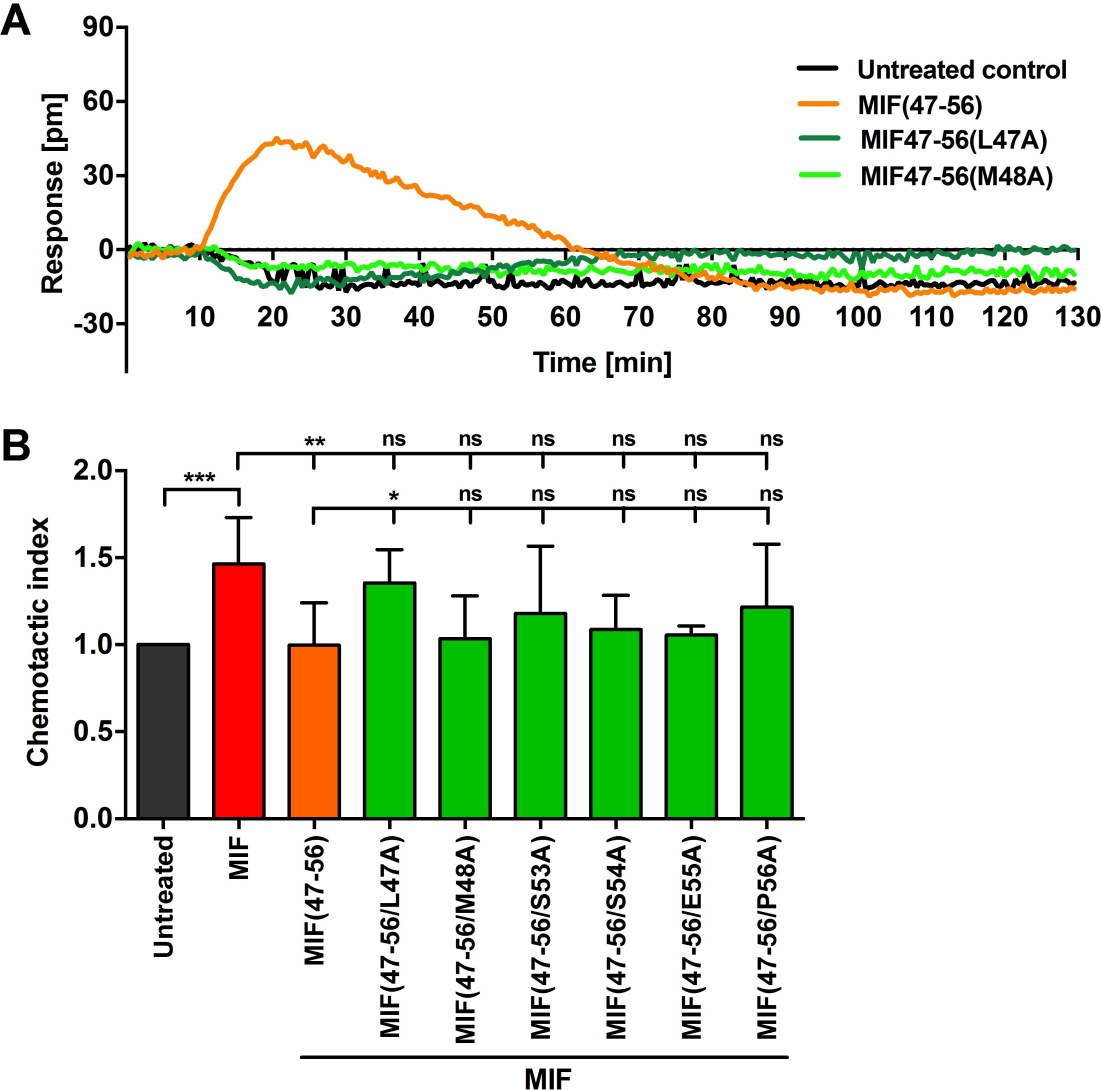
CXCR2 binding and inhibition properties of MIF(47–56) peptide analogues. A) Comparison of the binding capacity of MIF(47–56) and its Ala variants MIF(47–56/L47A) and MIF(47–56/M48A) (each applied at a concen‐tration of 500 μM) to CXCR2, as measured by DMR technology using HEK‐CXCR2 transfectants. B) Inhibitory capacity of MIF(47–56) and its Ala variants (5 μM) on monocyte chemotaxis elicited by human MIF (8 nM), as analyzed by a Transwell‐based assay device (*n*=3–11, mean±SD). Statistical significance is indicated: * *P*<0.05, ** *P*<0.01, *** *P*<0.001, ns=not significant.

The MIF(47–56) peptide Ala variants were screened for their CXCR2‐binding capacity by the DMR methodology using HEK‐CXCR2 transfectants compared to non‐transfected control cells (Figure 2A and Figure S1 in the Supporting Information). Treatment of HEK293‐CXCR2 transfectants with MIF(47–56/S53A), MIF(47–56/S54A), MIF(47–56/E5A), and MIF(47–56/P56A) showed changes in Δpm similar to that seen with MIF(47–56), thus suggesting that these substitutions do not alter the peptide's CXCR2‐binding response (Figure S1). Peptides MIF(47–56/E55A) and MIF(47–56/P56A) showed a somewhat “enhanced” activity, although this effect was not studied in more detail (Figure S1). Of note, the response was fully abrogated, when peptides MIF(47–56/L47 A) and MIF(47–56/M48 A) or a scrambled control sequence were tested, indicating that residues 47 and 48 are important for receptor binding (Figures 2A and S1). As expected, none of the peptides led to a response in non‐transfected control cells (Figure S1).

To further confirm the Ala‐scanning results and to investigate whether certain substitutions also impair the inhibitory potential of MIF(47–56), we tested the Ala variants in a Transwell leukocyte migration assay (Figure 2B). Leukocyte migration is stimulated by MIF and thus represents an atherosclerosis‐relevant *in vitro* assay system^[7a]^ suitable to screen inhibitory peptides. Monocyte chemotaxis is promoted by the MIF/CXCR2 axis. Primary human monocytes isolated from the peripheral blood mononuclear cell (PBMC) fraction of healthy donors were subjected to MIF‐induced chemotaxis in the presence of MIF(47–56) or its Ala variants. As shown previously,^[10]^ MIF‐induced monocyte migration was significantly inhibited by the parent peptide MIF(47–56). Of note, while inhibitory trends were observed for some Ala variants, MIF(47–56/L47A) with Leu‐47 exchanged for Ala, significantly failed to block MIF‐mediated monocyte chemotaxis (Figure 2B), overall confirming the DMR data and suggesting that MIF peptide position 47 is important for CXCR2 binding and inhibition of MIF/CXCR2‐mediated atherogenic activity. The replacement of Met‐48, which led to an ablated binding activity in the DMR assay, did not impair the inhibitory activity of MIF(47–56) on MIF‐induced chemotaxis. This apparent discrepancy could be due to microenvironmental conformational effects at the plasma membrane of HEK293 cells *versus* monocytes. Furthermore, although the apparent enhanced binding activity of the E55A and P56A analogues in the DMR assay is not significantly mirrored in the chemotaxis assay, a residue such as Glu‐55 might qualify for future substitutions to obtain improved analogues, but additional evidence from other assays is required to support this notion.

**Figure 3:**
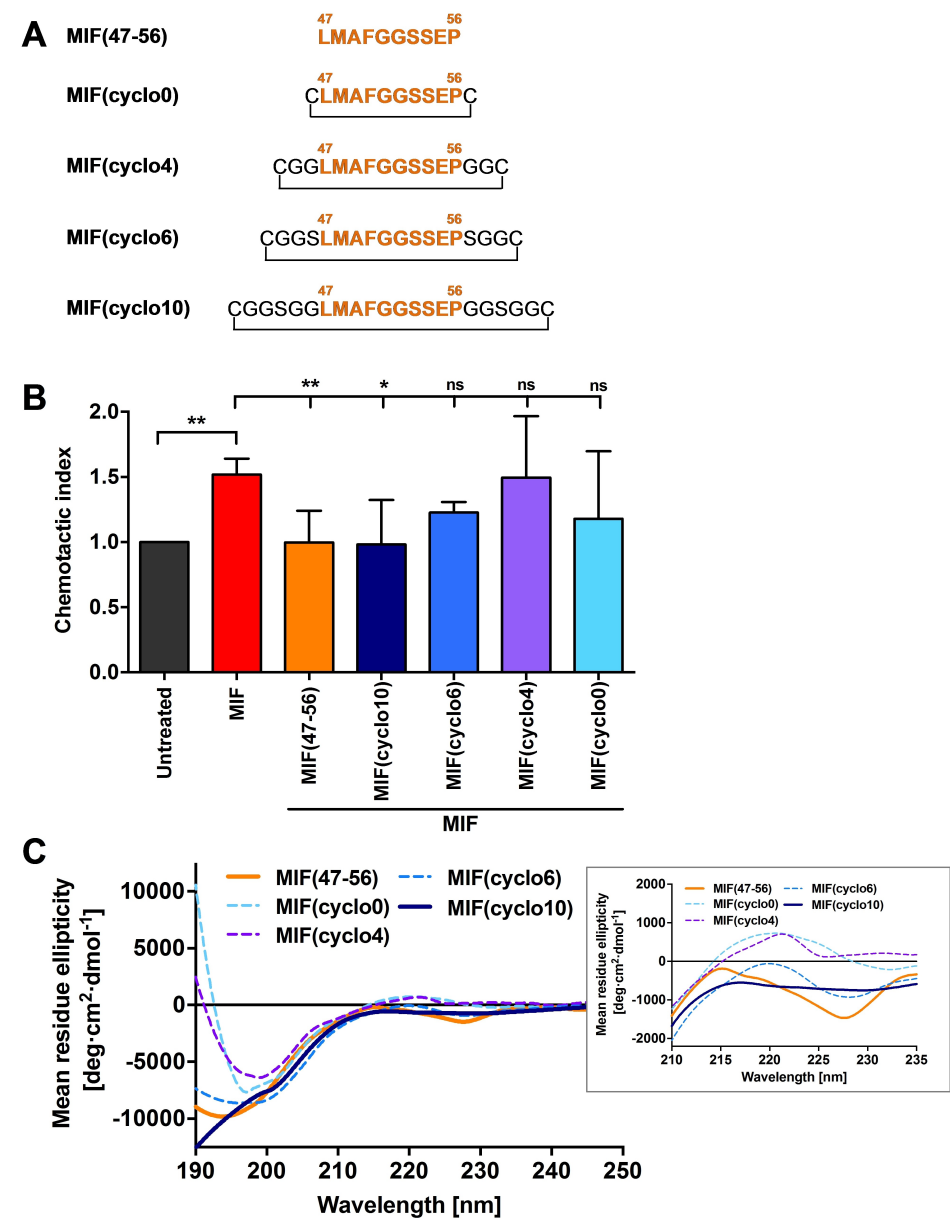
Screening of cyclic MIF(47–56) analogues for their inhibitory potential *in vitro* and characterization of their conformational properties. A) Overview of the screened cyclic MIF(47–56) analogues MIF(cyclo0), MIF(cyclo4), MIF(cyclo6), and MIF(cyclo10) and comparison to the linear parent peptide. The N‐like loop sequence 47–56 is highlighted in orange; amino acid sequences are depicted by the one‐letter code; cyclization through the disulfide bridge is indicated. B) Transwell‐based PBMC chemotaxis assay using MIF as the chemoattractant (*n*=4–11, mean±SD). Statistical significance is indicated: * *P*<0.05, ** *P*<0.01, ns=not significant. C) Circular dichroism (CD) spectra between 190 and 250 nm of the cyclic MIF(47–56) analogues in comparison to the linear peptide. Spectra were recorded three times, averaged, smoothed and are depicted as mean residue ellipticity (MRE). The inset is a close‐up of the spectra over the wavelength range 210–235 nm.

Based on this structure‐activity information, we wished to design cyclized variants of MIF(47–56). Cyclization may enhance proteolytic stability, but the conformational restriction imposed by peptide cyclization would have to preserve the CXCR2‐binding/MIF‐CXCR2‐inhibitory activity. Cyclic analogues of MIF(47–56) could provide a proof‐of‐concept for *in vivo* atherosclerosis blockers. Because of the crucial role of Leu47 that we had determined, we reasoned that the number of spacer residues inserted between the MIF‐derived peptide sequence and the terminal Cys residues to be introduced for cyclization may affect the properties of the cyclized variants. Overall, four cyclized peptides were designed with spacer lengths varied from 0 to 10 glycine/serine residues and termed MIF(cyclo0), MIF(cyclo4), MIF(cyclo6), and MIF(cyclo10), based on the number of glycine and serine residues in the spacer (Figure 3A). We further surmised that the stepwise addition of blocks of two glycines and an intermittent serine (e. g., in MIF(cyclo10)) would represent a reasonable compromise to jointly address aspects of spacer length, hydrophobicity, and conformational flexibility. To test the anti‐inflammatory/anti‐atherogenic capacity of the cyclized peptides, we analyzed them in the Transwell chemotaxis set‐up, studying their effect on MIF/CXCR2‐dependent chemotaxis responses of PBMCs (Figure 3B). We started out with a minimal cyclized variant of MIF(47–56), MIF(cyclo0), that only contained the two additional terminal cysteine residues needed for disulfide‐based cyclization. However, this analogue did not retain the inhibitory activity of MIF(47–56) on MIF‐mediated PBMC chemotaxis (Figure 3B).

Similarly, MIF(cyclo4), a cyclized analogue with an additional two glycine residues flanking each side of sequence 47–56, did not inhibit the chemotactic effect of MIF. Circular dichroism (CD) spectroscopy showed that in contrast to MIF(47–56), which adopts an overall random coil conformation with a pronounced minimum at 195 nm, MIF(cyclo0) and MIF(cyclo4) exhibited less random‐coil conformation and a significant positive band between 215 and 225 nm (Figure 3C, inset), overall indicative of a substantial degree of conformational restriction and a turn‐like conformational element.^[14]^ This result suggested that cyclic analogues with larger ring sizes and less conformational restriction could be required to retain the inhibitory function of MIF(47–56). In fact, analogue MIF(cyclo6), containing two additional spacer residues compared to MIF(cyclo4), displayed a CD spectrum that was more similar to that of the linear parent peptide MIF(47–56) (Figure 3C). However, the observed trend towards an inhibitory effect of MIF(cyclo6) on MIF‐mediated chemotaxis did not reach statistical significance (Figure 3B). We therefore further expanded the ring size by an additional four glycine residues to obtain MIF(cyclo10). Importantly, this analogue showed the full inhibitory activity of MIF(47–56) (Figure 3B) and exhibited a CD spectrum with similar random coil characteristics as the parent peptide (Figure 3C). Together, these data indicated that cyclization of MIF(47–56) needs to allow for a certain degree of conformational flexibility to be compatible with a preserved inhibitory capacity.

**Figure 4:**
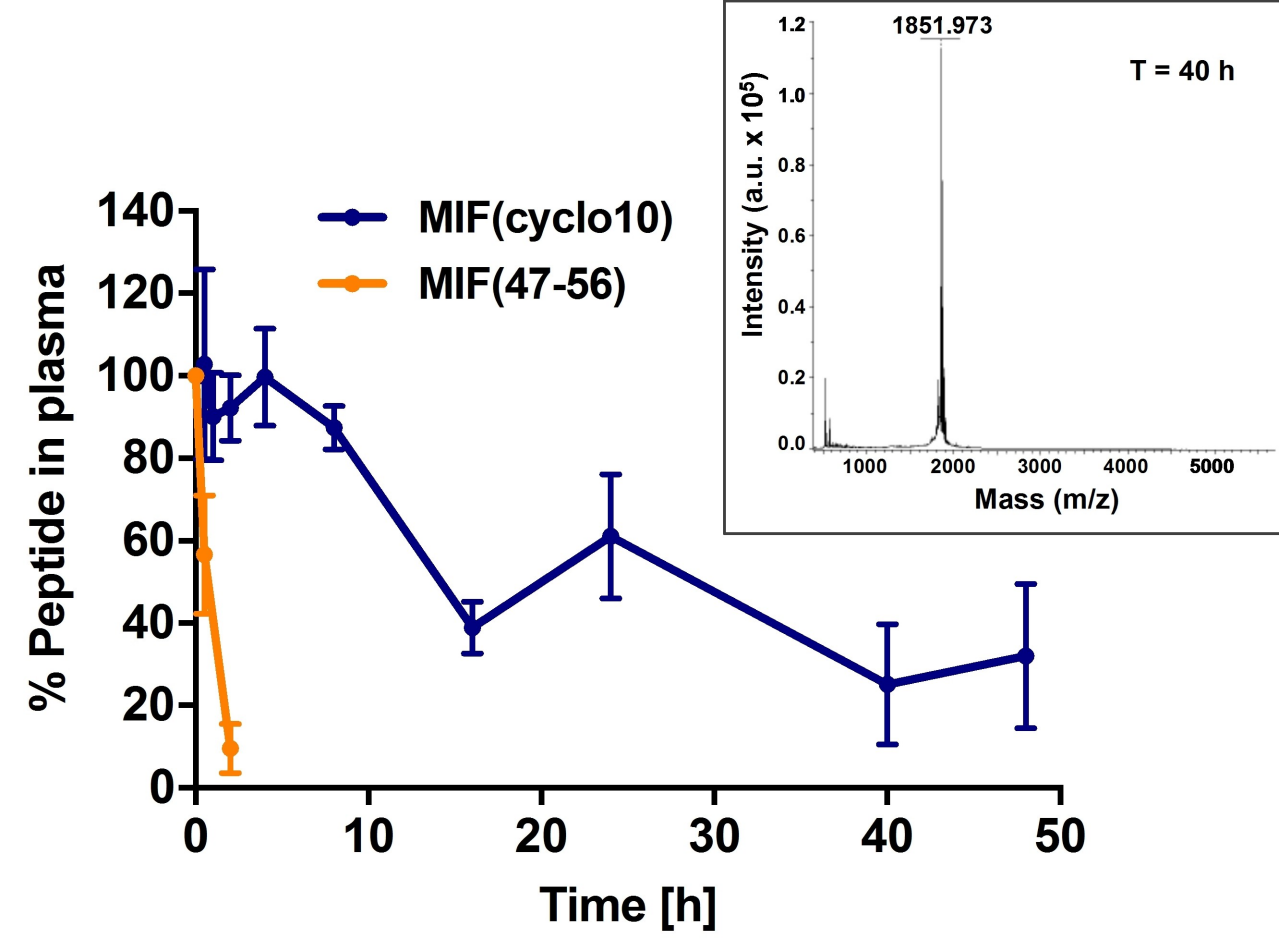
MIF‐derived cyclic peptide MIF(cyclo10) exhibits high proteolytic stability in human blood plasma. MIF(cyclo10) and the linear peptide MIF(47–56) were incubated in human plasma at 37 °C for the indicated time intervals, intact peptides were quantitated by C_18_ HPLC, and their molecular weights were verified by MALDI‐MS (see also Figure S2). The data shown are from three independent incubations, and error bars indicate mean±SD. Recovered intact peptide (% of total) is plotted over the various incubation time points. The inset shows the mass spectrum of the HPLC‐isolated peptide fraction after 40 h of incubation with plasma.

We next wished to determine the proteolytic stability of MIF(cyclo10) and compared it to that of the linear peptide MIF(47–56). MIF(cyclo10) or MIF(47–56) were incubated in human blood plasma for various time intervals and intact peptides were identified and quantified by HPLC and MALDI‐MS (Figures 4 and S2). As expected, the linear peptide was rapidly degraded in plasma (*t*1/2 ≈0.5 h; Figure 4). In contrast, degradation of MIF(cyclo10) was markedly delayed and appreciable amounts of intact MIF(cyclo10) were detectable up to 48 h (Figures 4 and S2). MIF(cyclo10) had thus a strongly improved proteolytic stability (*t*_1/2_>8 h), being more than 16‐fold resistant than MIF(47–56).

**Figure 5:**
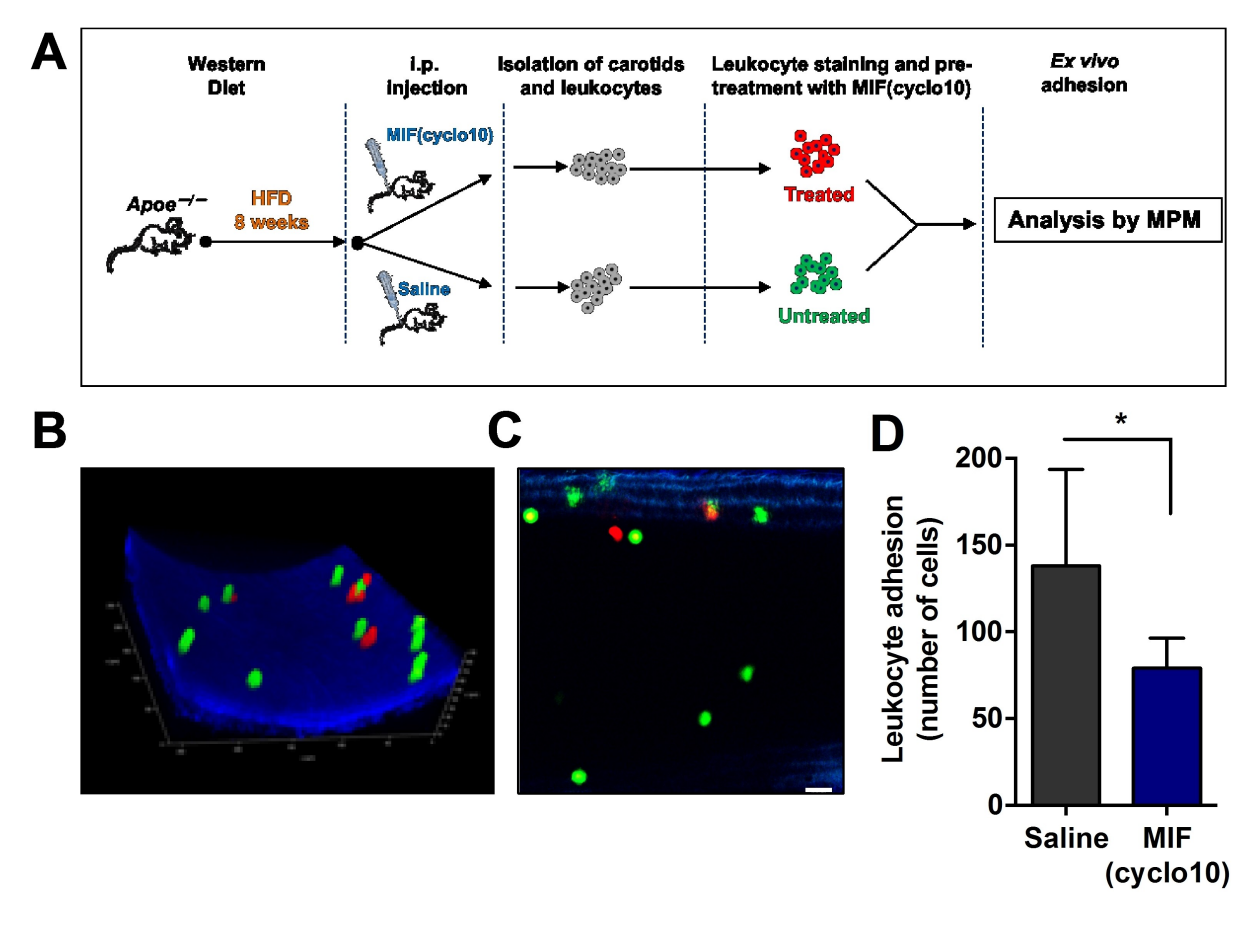
The MIF‐derived cyclic peptide MIF(cyclo10) blocks leukocyte adhesion to carotid arteries *in vivo/ex vivo*. A) Scheme indicating the experimental set‐up of the *ex vivo/in vivo* treatment regimen and leukocyte adhesion protocol in whole carotid arteries. B)–D) MPM analysis of adherent leukocytes after treatment with MIF(cyclo10). B)‐C) Example MPM images of adherent leukocytes to carotid arteries after treatment (red) or vehicle (green); B) 3D acquisition of a *z*‐stack series; C) representative 2D image; scale bar: 20 μm. D) Quantification of adherent leukocytes after treatment with MIF(cyclo10). Adherent cells under flow were quantified by MPM *in situ*. The data shown are mean±SD and are derived from four independent MIF(cyclo10)‐treated and four saline‐treated mice. Adhesion data were obtained on five (MIF(cyclo10)) and seven (saline) independent carotid arteries. Four of the prepared carotids did not inflate and could not be used for the adhesion experiments. Statistical significance is indicated: * *P*<0.05.

As the cyclic peptide MIF(cyclo10) was effective in blocking MIF‐mediated leukocyte recruitment activity *in vitro* and exhibited a favorable proteolytic stability in blood plasma, we finally asked whether this peptide would exhibit anti‐atherogenic activity in an atherosclerosis‐relevant *ex vivo/in vivo* experimental setting. MIF(cyclo10) was intraperitoneally injected into pro‐atherogenic *Apoe* ^*–/–*^ mice that had been on cholesterol‐rich high‐fat diet (HFD) for 8 weeks, administering a dose of 50 μg/day (i. e. 2.5 mg/kg/day) for three days before carotid arteries were prepared. After further preincubation with MIF(cyclo10), whole‐mount arteries were perfused with MIF(cyclo10)‐pretreated leukocytes and adherent leukocytes enumerated in an *ex vivo* adhesion set‐up under physiologically relevant flow conditions by multiphoton laser‐scanning microscopy (MPM; Figure 5A). Treatment with MIF(cyclo10) led to a significant reduction in arterial leukocyte adhesion compared to the vehicle‐treated control group (Figure 5B–D), demonstrating that MIF(cyclo10) acts as a leukocyte recruitment inhibitor *in vivo/ex vivo*.

Together, our results highlight that designed N‐loop‐containing MIF‐derived peptides that are conformationally constrained by disulfide cyclization such as MIF(cyclo10) could be promising inhibitors of MIF's disease‐promoting pro‐inflammatory and atherogenic activities. While the proof‐of‐principle obtained in our current study was based on biochemical and relevant cellular *in vitro* assays, as well as an *in vitro* proteolytic stability assay in human plasma and a short‐term *ex vivo* model of atherogenic leukocyte recruitment, it is tempting to hypothesize that MIF(cyclo10) or further improved analogues thereof could also confer protection in chronic models of atherosclerosis that would encompass treatment regimens over several weeks or months. We further conclude that MIF(cyclo10) is a cyclized analogue of MIF(47–56) that shows strongly improved proteolytic stability, while retaining the inhibitory activity of its linear precursor MIF(47–56). MIF(cyclo10) is thus an attractive peptide lead for drugs targeting pathogenic MIF‐mediated inflammation. In fact, while peptide‐based drugs are sometimes viewed to be inferior to antibody or small molecule approaches regarding aspects of plasma stability and pharmacokinetics, available peptide design and synthesis strategies of introducing unnatural amino acids, d‐amino acids, pseudo‐peptide bonds, and cyclic constraints offer numerous options to devise highly active and reasonably stable peptide drugs.^[14]^ Moreover, peptide approaches are favorable due to low synthesis/production costs, while offering higher target affinities and specificities compared to small molecule drugs.^[14b]^

Furthermore, the tailored MIF/CXCR2 interface‐targeting approach as afforded by MIF(cyclo10) or further optimized analogues thereof could provide additional beneficial features compared to established antibody or small molecule‐based MIF‐targeting strategies. MIF generally is a pro‐inflammatory and pro‐atherogenic mediator, but it also has been appreciated that it may have context‐ and tissue‐specific dual roles with both protective and disease‐exacerbating activity.[^6^, ^8^, ^15^] For example, in the acute setting of myocardial ischemia/reperfusion (I/R) injury, MIF is cardioprotective in the ischemic and early reperfusion phase, while its inflammatory, disease‐exacerbating activities predominate in the mid‐late and post‐reperfusion phase, in which MIF contributes to inflammatory leukocyte infiltration processes.[^6^, ^8^, ^16^] Anti‐MIF antibodies or MIF‐directed small molecules have been found to block the MIF/CD74 axis, which is critical to MIF's cardioprotective effect in the early ischemic phase, and would thus be less suitable in such a disease setting. Conversely, in addition to representing a promising MIF/CXCR2‐directed approach in the chronic setting of atherosclerosis and inflammation, it could be speculated that a MIF(cyclo10)‐based MIF/CXCR2‐specific targeting approach could be tailored to specifically target MIF's exacerbating effect in the post‐I/R setting. Similarly, cyclic peptide‐based MIF/CXCR2 targeting approaches could be advantageous compared to antibody‐ or small molecule‐based CXCR2‐targeting strategies such as reparixin that would also interfere with physiological or angiogenic effects of the CXCL8/CXCR2 axis.

## Conclusion

Based on the N‐like loop sequence 47–56 of the atypical chemokine MIF that is a determinant of the MIF/CXCR2 interface and competes with MIF at the receptor, we have identified MIF(cyclo10), a macrocyclic peptide inhibitor of MIF's disease‐promoting pro‐inflammatory and atherogenic activities that exhibits pronounced proteolytic stability in human plasma. A MIF/CXCR2 interface‐specific strategy could be of value in several diseases, where MIF's role is dichotomous or phase‐specific and/or where beneficial activities of the CXCL8/CXCR2 pathway need to be maintained.

## Experimental Section

**Cells and cell culture reagents**: HEK293‐CXCR2 transfectants were initially provided by Dr. Ben‐Baruch (Tel Aviv University, Israel) and have been reported on before.^[7a]^ Briefly, cells were cultured in DMEM/F‐12 (1 : 1) supplemented with GlutaMAX^TM^, 10 % fetal calf serum (FCS) and 1 % penicillin/streptomycin (P/S). PBMCs were isolated from buffy coats provided by the Department of Transfusion Medicine at RWTH Aachen University Hospital applying Ficoll gradient centrifugation. Primary CD14^+^ monocytes were enriched using immunomagnetic separation (Miltenyi Biotec, Bergisch Gladbach, Germany). Monocytes were cultured in RPMI‐1640 supplemented with 10 % FCS and 1 % P/S. Cell culture reagents were purchased from Thermo Fisher Scientific (Invitrogen). Cells were cultivated at 37 °C and 5 % CO_2_. Biologically active, endotoxin‐free recombinant human MIF (rMIF) was expressed and purified as described previously.[^7a^, ^17^] Fluorescent labeling of MIF was performed with the Microscale Protein Labeling Kit from Invitrogen‐Molecular Probes (Karlsruhe, Germany; Alexa‐488/A30009) according to the manufacturer's protocol.

**Peptides**: Linear MIF‐derived peptides were generated by standard Fmoc‐based solid phase peptide synthesis (SPPS) in their N‐terminally acetylated and C‐terminally amidated forms and purified by reversed‐phase (RP)‐HPLC as previously described.^[18]^ 5(6)‐Carboxyfluorescein (Fluos) N‐terminally labeled peptides were synthesized as described.^[19]^ Peptide purity was verified by matrix‐assisted laser desorption ionization mass spectrometry (MALDI‐MS). Cyclic peptide derivatives MIF(cyclo0), MIF(cyclo4), MIF(cyclo6), and MIF(cyclo10), containing terminal cysteines and different numbers of glycine and serine spacer residues to tailor ring size, were custom‐synthesized and purified (>96 % purity grade) by American Peptide Company (APC), Inc./Bachem (Bubendorf, Switzerland), or as described previously,^[20]^ applying standard disulfide‐based oxidation procedures. All tested peptides were readily soluble in the aqueous solutions used in the assays as verified by visible inspection, centrifugation‐based precipitation analysis, and in the case of MIF(47–56) and MIF(cyclo10) also by CD concentration dependence studies between 5 and 100 μM, which showed no concentration dependence of the CD spectra. The sequence of the scrambled control peptide of MIF(47–56) is SFESGPAGML.

**Fluorescence spectroscopy**: Fluorescence spectroscopic titrations were performed on a Jasco FP‐6500 spectrofluorometer. Alexa Fluor‐488‐labelled rMIF (Alexa‐MIF) was reconstituted in 20 mM sodium phosphate buffer (pH 7.2). MIF(47–56) was dissolved in 1,1,1,3,3,3‐hexafluoropropan‐2‐ol (HFIP) and substocks were prepared following serial dilutions in the same solvent. Alexa‐MIF and MIF(47–56) were mixed in a quartz cuvette to adopt final experimental conditions of 10 mM sodium phosphate (pH 7.4) and 1 % HFIP. The final concentration of Alexa‐MIF was 10 nM and its mixtures with MIF(47–56) were prepared at ratios of 1 : 0.1, 1 : 1, 1 : 10, 1 : 100, 1 : 250 and 1 : 1000. The excitation wavelength was set at 492 nm and fluorescence emission spectra were recorded between 500 and 600 nm within 2–3 min after the preparation of the mixtures at room temperature (RT).

**Structural models and structure prediction**: Three‐dimensional structures of human MIF and human CXCL8 as well as the predicted structure of human CXCR2 were visualized using the PyMOL Molecular Graphics System, version 1.8.2.2 (Schrödinger, LLC). The structures represent the Protein Data Bank files for MIF (PDB ID: 3DJH), CXCL8 (PDB ID: 6 N2 U) or our molecular docking results. To visualize CXCR2, its 3D structure was modeled based on its amino acid sequence (UniProt entry P25025) via the Phyre^2^ webserver, using the intensive modeling mode.^[11]^ *Molecular docking*. To visualize the interaction of monomeric MIF with CXCR2, we used the PatchDock server.^[21]^ The residues corresponding to MIF's N‐like loop as well as CXCR2’s N‐terminal, ECL1, and ECL2 regions were chosen as interaction sites. Docking results were refined using FireDock.^[12]^

**Receptor binding assay**: Interactions between MIF(47–56) and CXCR2 were analyzed by a receptor binding assay using HEK293 cells stably transfected with human CXCR2 (HEK293‐CXCR2) in comparison with non‐transfected HEK293 cells (HEK293‐WT). Fluos‐labeled MIF(47–56) was reconstituted at a concentration of 1 mM in phosphate‐buffered saline (PBS) and used at a final concentration of 3.7 or 7.4 μM. Five ×10^5^ cells were incubated with Fluos‐MIF(47–56) on ice for 2 h. Cells were washed and the binding of Fluos‐MIF(47–56) to cell surface‐expressed CXCR2 assessed by measuring the mean fluorescence intensity (MFI) of the cells by flow cytometry using a Becton Dickinson (BD) FACS Verse (Heidelberg, Germany). The MFI signal of untreated HEK‐WT cells was set to 1 and the intensities upon incubation with Fluos‐MIF(47–56) were normalized accordingly.

**Label‐free dynamic mass redistribution**: Dynamic mass redistribution (DMR) analysis^[22]^ was performed according to Perkin Elmer's instructions for label‐free DMR measurements. Briefly, cells were seeded at a density of 40 000 cells per well of an EnSpire label‐free 96‐well fibronectin‐coated cell assay microplate (PerkinElmer). Post‐seeding, the cells were equilibrated at RT for 30 min and cultivated overnight in serum‐containing DMEM/F‐12 medium. The next day, the cells were washed four times with assay buffer containing 20 mM HEPES and 1 % DMSO in HBSS (pH 7.4), using an aspiration wand leaving a residual volume of 35 μL. The plate was equilibrated at RT for 2 h. MIF‐derived peptides were reconstituted at a concentration of 5 mM in PBS and further diluted to a working concentration of 2.5 mM in assay buffer. Before adding the peptides, baseline measurements were performed for 10 min (30 s intervals) using the EnSpire Label‐free Multimode Plate Reader (PerkinElmer). Then, 20 μL of the respective peptides were added to the cells at a final concentration of 500 μM followed by real‐time measurements over a time period of 40 min (30 s intervals). The signal output represents a cumulative response overlay of all cells.

**Transwell migration assay**: The inhibitory potential of the MIF‐derived peptide analogues was measured by Transwell chemotaxis assay using CD14^+^ monocytes isolated from PBMCs or the entire PBMC fraction, as previously described.^[7a]^ Briefly, PBMCs from buffy coats or CD14+ monocytes isolated from PBMCs were diluted in RPMI1640 supplemented with 0.5 % bovine serum albumin (BSA) at a density of 1×10 cells/mL. Cells were placed into the upper chamber of a 24‐well cell culture inserts with 5 μm pore size (Corning). 8 nM full‐length MIF served as chemoattractant and was pre‐incubated with or without 5 μM of the respective peptides and applied to the lower chamber. After a 3 h migration interval at 37 °C (5 % CO_2_), cells were collected and counted via a CASY Cell Counter (OLS, OMNI Life Science, Bremen, Germany).

**Circular dichroism spectroscopy**: CD spectra were recorded on a Jasco J‐715 spectropolarimeter in a wavelength range between 190 and 250 nm. Spectra were measured at 0.1 nm intervals and a response time of 1 s. Peptide solutions were freshly prepared at a final concentration of 10 μM in 10 mM sodium phosphate buffer (pH 7.4), vortexed and transferred to quartz cuvettes. Measurements were performed at room temperature. Dynode voltage values were below 1000 and did not interfere with the CD measurements. Each spectrum was measured three times, averaged and smoothed after background subtraction of the buffer. Spectra are presented as mean residue ellipticity (MRE).

**Proteolytic stability assay**: Proteolytic stability studies of peptides were performed following a previously described experimental protocol.[^20^, ^23^] Briefly, peptides were incubated (200–500 μg/mL) in human plasma, isolated from the blood of healthy volunteers by routine methods using citrate (10 mM, pH 7.0) as an anti‐coagulant, at 37 °C for various time intervals. At the end of each interval, a stop solution (1 : 1) containing aqueous trichloroacetic acid (TCA, 10 %, *v*/*v*) was added and the samples kept on ice for 10 min. Samples were centrifuged at 20 000 g for 4 min, the supernatants retrieved and mixed 1 : 2 with a solution of 80 % HPLC buffer B (0.05 % (*v*/*v*) TFA in 90 % (*v*/*v*) CH_3_CN in water) and 20 % HPLC buffer A (0.058 % (*v*/*v*) TFA in water). Samples were analyzed by RP‐HPLC (detection at 214 nm) using a Nucleosil 100 C18 column (Grace; length 33 mm, i.d. 8 mm, 7 μm particle size).^[24]^ The flow rate was 2 mL/min and the samples were analyzed with an elution program consisting of 2 steps; first step: 10 % HPLC buffer B in buffer A (1 min) and second step: a gradient from 10 to 90 % HPLC buffer B in buffer A (6 min). Peaks were collected, immediately frozen, lyophilized, and their molecular weights determined by MALDI‐MS. The HPLC peak areas were used to quantify remaining peptides in plasma.

***In vivo/ex vivo*** **monocyte recruitment assay**: Mice were housed under standardized and specific pathogen‐free conditions at the Center for Stroke and Dementia Research (CSD), Munich, Germany, with free access to food and water. Mice were between 7–8 weeks of age and were on C57BL/6 background. *Apoe* ^*–/–*^ mice were obtained from Charles River Laboratories (Sulzfeld, Germany) and backcrossed within the CSD animal facility. Mice received a high‐cholesterol diet (“Western diet”, 0.2 % cholesterol, TD88137, SNIFF Spezialdiäten GmbH, Soest, Germany) for 6 weeks prior to an intraperitoneal injection with peptide MIF(cyclo10) or an equivalent volume of saline. During the three days before carotid artery preparation, injections were performed at a dose of 50 μg per injection and day. Mouse experiments were approved by the Animal Care and Use Committee of the local authorities and performed in accord with the animal protection representative at CSD (animal ethics approval ROB‐55.2‐2532.Vet_02‐18‐40). After the treatment, carotid arteries were explanted and leukocytes isolated and stained with CMFDA‐ or CMPTX‐fluorescent dyes (Invitrogen) according to the manufacturer's recommendation and as described previously.^[25]^ Leukocytes and isolated carotid arteries were pre‐incubated with 3 μM MIF(cyclo10) or saline as vehicle control at 4 °C for 1 h. Finally, carotid arteries were perfused with a mixture of stained leukocytes for 30 min and adherent cells visualized and quantified by MPM using a Leica TCS SP8 MP DIVE instrument.

**Statistical analysis**: Data were analyzed with GraphPad Prism Version 6.0 and 7.0. After testing for normality distribution with the Shapiro‐Wilk or D′Agostino & Pearson normality test, statistical significance was calculated by unpaired Student's t‐test or one‐way ANOVA (parametric tests) or Kruskal‐Wallis‐ or Mann‐Whitney‐U‐test (non‐parametric tests) as appropriate. Differences between groups with *P*<0.05 were considered statistically significant.

## Supporting information

As a service to our authors and readers, this journal provides supporting information supplied by the authors. Such materials are peer reviewed and may be re‐organized for online delivery, but are not copy‐edited or typeset. Technical support issues arising from supporting information (other than missing files) should be addressed to the authors.

Supplementary
