# Supplementary material for: A MIF‐Derived Cyclopeptide that Inhibits MIF Binding and Atherogenic Signaling via the Chemokine Receptor CXCR2
Source: Chembiochem. 2020 Nov 30;22(6):1012–9. doi: 10.1002/cbic.202000574 (PMC8049018; doi:10.1002/cbic.202000574)
Supplement: Supplementary file 1 — Supplementary [file CBIC-22-1012-s001.pdf]

ChemBioChem

Supporting Information

A MIF-Derived Cyclopeptide that Inhibits MIF Binding and Atherogenic Signaling via the Chemokine Receptor CXCR2

Christine Krammer, Christos Kontos, Manfred Dewor, Kathleen Hille, Beatrice Dalla Volta, Omar El Bounkari, Karin Taş, Dzmitry Sinitski, Markus Brandhofer, Remco T. A. Megens, Christian Weber, Joshua R. Schultz, Jürgen Bernhagen,* and Aphrodite Kapurniotu*

SUPPLEMENTARY METHODS

Label-free dynamic mass redistribution: Dynamic mass redistribution (DMR) analysis[19] was performed according to Perkin Elmer's instructions for label-free DMR measurements. Briefly, cells were seeded at a density of 40,000 cells per well of an EnSpire label-free 96-well fibronectin-coated cell assay microplate (Perkin Elmer, Rodgau, Germany). Post-seeding, the cells were equilibrated at room temperature (RT) for 30 min and cultivated overnight in serum-containing DMEM/F-12 media. The next day, the cells were washed four times with assay buffer containing 20 mM Hepes and 1% DMSO in HBSS (pH 7.4), using an aspiration wand leaving a residual volume of 35 µL. The plate was equilibrated at RT for 2 h. MIF-derived peptides were reconstituted at a concentration of 5 mM in PBS and further diluted to a working concentration of 2.5 mM in assay buffer. Before adding the peptides, baseline measurements were performed for 10 min (30 sec intervals) using the EnSpire Label-free Multimode Plate Reader (Perkin Elmer). Then, 20 µL of the respective peptides were added to the cells at a final concentration of 500 µM followed by real-time measurements over a time period of 40 min (30 s intervals). The signal output represents a cumulative response overlay of all cells.

SUPPLEMENTARY FIGURES

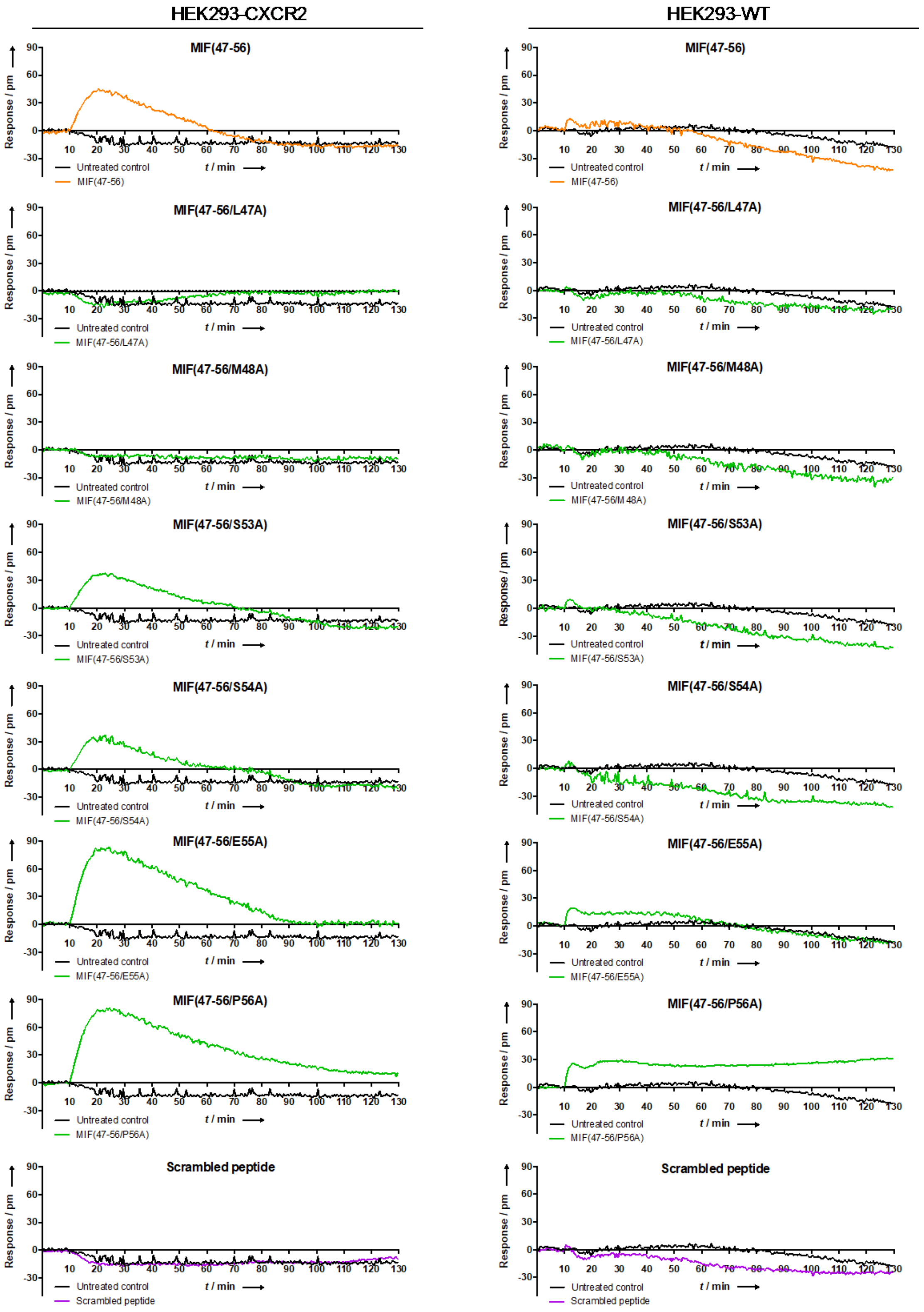

Figure S1. Screening of MIF(47-56) Ala variants for their binding capacity to CXCR2 by DMR technology using HEK-CXCR2 transfectants (left) compared to non-transfected control cells (WT, right). A scrambled sequence of MIF(47-56) was used to determine unspecific binding (bottom). All peptides were applied at a concentration of 500 µM).

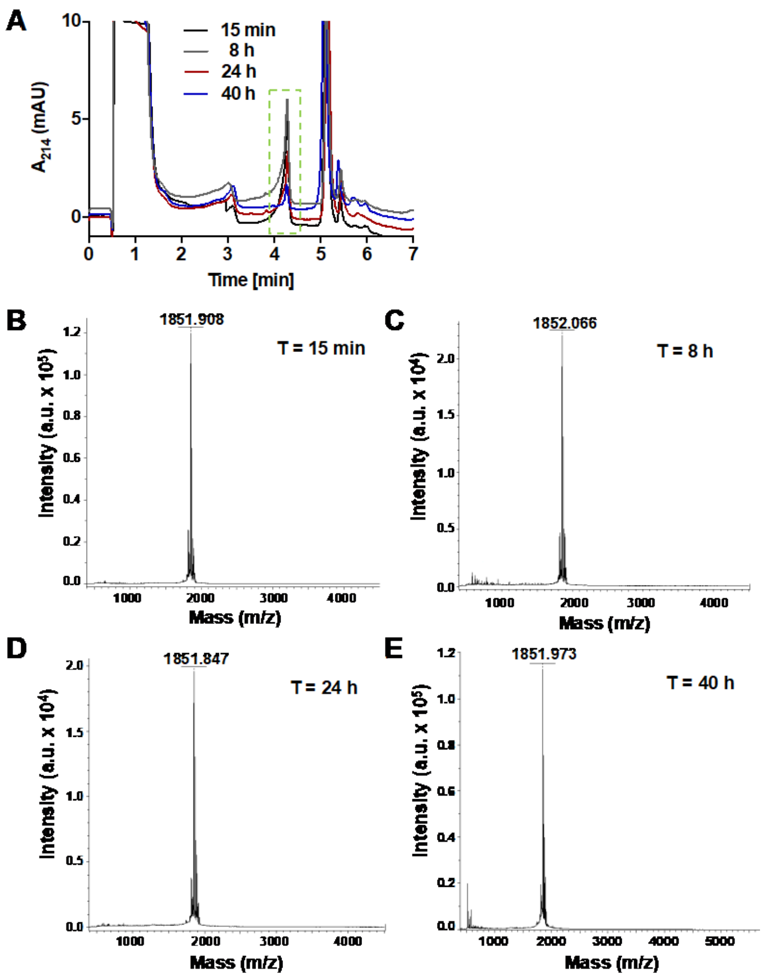

Figure S2. MIF-derived cyclic peptide MIF(cyclo10) exhibits high proteolytic stability in human blood plasma. The HPLC chromatogram (A) and the mass spectra of recovered peptide MIF(cyclo10) at the various time points (B-E) are shown. MIF(cyclo10) was incubated (225 µg/mL) in human plasma at 37°C for the indicated time intervals, recovered peptides quantitated by C18 HPLC and their molecular weights verified by MALDI-MS (see also Figure 4). (A) HPLC chromatograms with the chromatograms of the four incubation times indicated by color code. The peaks at 4.2 min that are marked by a dotted green box are the peptide fractions that were isolated and subjected to MALDI-MS. The peaks at 5.1 min represent remaining (non-precipitated) bulk plasma proteins. The chromatograms shown are representative of three independent incubations. (B-E) Mass spectra of the isolated peptide peaks at 4.2 min according to (A), representing the different incubation intervals (B, 15 min; C, 8 h; D, 24 h; E, 40 h).
